# Determinants of health- related quality of life (HRQoL) among deaf and hard of hearing adults in Greece: a cross-sectional study

**DOI:** 10.1186/s13690-018-0304-2

**Published:** 2018-10-08

**Authors:** Dialechti Tsimpida, Daphne Kaitelidou, Petros Galanis

**Affiliations:** 1 Manchester Centre for Audiology and Deafness (ManCAD), Division of Human Communication, Development and Hearing, School of Health Sciences, Faculty of Biology, Medicine and Health, University of Manchester, Manchester Academic Health Science Centre, A4.01 Ellen Wilkinson Building, Oxford Road, Manchester, M13 9PL UK; 2 Center for Health Services Management and Evaluation, Department of Nursing, National & Kapodistrian University of Athens, Athens, Greece

**Keywords:** Deaf, Hard of hearing, Deafness, Health-related quality of life, Determinants

## Abstract

**Background:**

Hearing loss is an important public health issue, since it has a very negative impact on peoples’ lives, irrespective of the age at which it develops. However, globally there is a noticeable lack of epidemiological data for health outcomes for people who are deaf and hard of hearing. In Greece, people with hearing disabilities are systematically not included in health policy and planning processes, despite there being a marked tendency for global efforts aimed at improving their quality of life.

**Methods:**

The sample consisted of 140 adults with hearing loss (86 d/Deaf and 54 hard of hearing) and 97 normal hearing as the control group. We run data collection from April to June 2015, using the Greek version of the 36-Item Short Form Health Survey (SF-36v2). Socio-demographic and characteristics about non-medical determinants of health (tobacco and alcohol consumption levels, BMI and physical activity).were also collected and were analysed as possible determinants. Data analysis included bivariate and multivariate analyses such as linear regression models.

**Results:**

Multivariate analyses identified that in all the SF-36v2 dimensions, the scores among deaf people were lower than those with normal hearing. Determinants included the hearing loss degree, educational level, body mass index, levels of physical activity, and alcohol consumption levels, while the variable “number of family members per household” was inversely associated with physical health summary scale score.

**Conclusions:**

Improving knowledge of the health-related determinants that affect quality of life for the population with hearing loss is an important step in designing targeted services and interventions. In light of these findings, a special effort must be made to ensure the wellbeing of this population.

## Background

Hearing impairment, deafness or hearing loss is a partial or total inability to hear: it may be ranked as mild, moderate, moderately severe, severe or profound. Impairment may result from genetic causes, complications at birth, certain infectious diseases, chronic ear infections, the use of particular drugs, exposure to excessive noise and ageing [1]. Although the consequences of hearing loss are never obvious, hearing loss is a major global health challenge, as over 5% of the world’s population – 360 million people – has disabling hearing loss (hearing threshold of 41 dB or greater in the better ear) [1]. Hearing loss –irrespective of the age at which it develops – has serious consequences for interpersonal communication, psychosocial well-being and individual quality of life [2, 3]. In most countries, hearing loss is projected to be among the top ten burdens of disease by 2030, with the associated detrimental social and economic effects, which will be greater in low-and middle-income countries [2].

Despite the seriousness of this public health issue, there is a noticeable lack of epidemiological data for health outcomes for people who are deaf and hard of hearing [4]. However, the few studies that have focused specifically on this topic [5–11] showed that while the implications of hearing loss differ from person to person, it consistently has a negative impact on peoples’ lives across numerous quality of life measures, including mental health, social functioning and general health.

In Greece, people with hearing disabilities are systematically excluded from health policy and planning processes, despite there being a marked tendency for global efforts aimed at improving their quality of life [4]. Furthermore, due to the severity of the economic downturn that Greece is facing, government grants for Greek Sign Language (GSL) interpretations have significantly reduced. That means that those who communicate via GSL have been forced to reduce their quality of life, due to communication barriers they face. From 2011 to date, the GSL users have the right to 25 h of free interpretation per year; whereas prior to 2011, the State fully paid interpretation costs for all their communication needs, and the available interpretation hours per person were unlimited. The contribution of GSL interpretation in the quality of life of Deaf/HH GSL users is enormous, since only via the presence of an interpreter do these people enjoy interactive and effective communication in all aspects of their life, covering all their communications needs, including use of and access to healthcare services (hospitals and clinics) [12]. In case GSL users consume their 25 free hours of interpretation, they then have to pay the interpretation costs making private payments, which are significantly higher when related to health issues. This is an important barrier for this population in accessing health services, with varying implications on their health-related quality of life (HRQoL).

Identifying determinants of the health-related quality of life of deaf and hard of hearing people is of great importance so as their health status can be improved. This study aims to fill a gap in the literature, by investigating the epidemiological profile of deaf and hard of hearing, so thatthe futurepolicy on their healthcare needs would be evidence-based. The objectives of this study included an assessment of the health-related quality of life of deaf and hard of hearing adults (aged 18–65), and the investigation of the factors that affect it.

## Methods

### Participants

A cross-sectional study was conducted, and the study population consisted of 140 young and middle-aged adults (18–65 years) with hearing loss (86 d/Deaf and 54 hard of hearing) and 97 hearing participants. There are several groups included within the broad “deaf and hard of hearing” category, as the population with hearing loss consists of subgroups with distinctly different cultural and communicational characteristics, which need to be separately examined. Factors that must be considered with Deaf/HH sub-populations include a) degree of hearing loss, b) age of onset of loss (pre-lingual/post-lingual), c) preferred language, and d) psychological issues [13]. Following a convention proposed by James Woodward [14], we use the lowercase *deaf* when referring to the audiological condition of not hearing, and the uppercase *Deaf* when referring to a particular group of deaf people who share a language – (GSL) – and a culture. The members of this group have adopted the GSL in their daily life, they use it as primarily for their communication and hold a set of beliefs about themselves and their connection to wider society. We distinguish them from, for example, those who find themselves losing their hearing because of illness, trauma or age; although those people share the condition of not hearing, they do not have access to the knowledge, beliefs and practices that make up the culture of Deaf people. Additionally, the distinction between the definition of “deaf” and “hard of hearing” is often given under different criteria, as it has strong ties with the individuals’ ideological positions about education, social inclusion and/or rehabilitation of people with hearing loss [13].

In this study, the participants with hearing loss were categorised by combination of cultural self-identification, and their preferred method of communication, in order to self-identify themselves in one of the following five categories; a classification that fits best with the population with hearing loss in Greek culture.

1. Deaf – Persons referred to as *Deaf* (upper case D) typically belong to the Deaf Community and use GSL as a primary language [14].
2. deaf – Persons referred to as *deaf* (lower case d), that do not consider themselves members of the Deaf Community, although they may be severely or profoundly deaf, strive to identify themselves with hearing people and regard their hearing loss solely according to biomedical model of health, using non-signing communication [15].
3. Hard of hearing that communicate in terms of auditory-verbal approach (emphasis is given on strengthening the auditory channel through listening only – before any visual information is presented) [16].
4. Hard of hearing who communicate via lip-reading (a technique of understanding speech by visually interpreting the movements of the lips, faces and tongue) [17].
5. Hard of hearing who communicate using GSL as a preferred method of communication [14].

Regarding the sampling method, there is no accurate census of deaf and hard of hearing in Greece since members of the subcategories of this population are extremely difficult to be identified, except from the members of the first subcategory who are registered in deaf clubs- Thus, the probability or random sampling was not feasible and therefore a convenience sampling technique was used. In regards to the deaf and hard of hearing participants that communicate via GSL, the recruitment was done by visiting the 5 Deaf Clubs-members of the Hellenic Federation of the Deaf which are located in Attica (4 in Athens and one in Korydallos). In addition, the Hellenic Federation of the Deaf embedded a link of an online survey in their website. This helped the wide distribution of the questionnaire and to reach the selected population, as deaf and hard of hearing individuals in Greece use this website to get local news and information, even if they do not communicate via sign-language. We also had placed a link on social networks, to provide also quick access to participants with similar demographics that had normal hearing, according to their answers in self-reported hearing difficulties.

### Materials

The questionnaire consisted of three sections. The first section explained the purpose of the questionnaire and provided a contact name and address for any enquiries regarding the study. The second and third section consisted of the 36-item Short Form Health Survey (SF-36v2), and questions on standard socio-demographic data such as gender, age, marital status, number of family members per household, complete educational status, work status, income and non-medical determinants of health (tobacco and alcohol consumption, BMI and physical activity). The SF-36 Health Survey Version 2, for the measurement of the perceived health-related quality of life, is a widely used questionnaire. The SF-36v2 Health Survey health domain scales are: Physical Functioning, Role-Physical, Bodily Pain, General Health, Vitality, Social Functioning, Role-Emotional, Mental Health. Higher scores across each subscale indicate greater HRQoL. The psychometrically-based physical component summary and mental component summary scores are also provided. The SF-36 has been translated and its psychometric properties tested for a Greek population by Pappa et al. [18].

A pilot study with 6 deaf adults was carried out, in order to check the comprehensibility of the questionnaire, as for the Sign Language users the Greek language is being perceived as a second language, and it is being taught to the Schools of the Deaf along with the GSL, which is their first language. In addition, they are obligated to learn only the written form of the Greek language and not the oral form according to the Greek Law N^o^3699/2008. The 6 pilot subjects were representative of the overall Deaf signers that were enrolled into this project, as they represent them unanimously on a regular basis in the Community, and they have a deep understanding of their educational and other needs. Face validity of the questionnaire was very good and no significant corrections were made.

### Procedure

The data collection was done in person, so that the questions were presented in GSL from the first author (TD) when it was necessary, in order to improve accessibility and minimise the language barrier for Deaf, as they completed a lengthy written questionnaire in a second language. The completion of the questionnaire by the deaf and hard of hearing non-users of GSL and by the hearing participants was done in person, following a convenience sampling technique.

All participants were informed about the aim and procedures of this study (additionally in GSL by TD if it was necessary) and gave their consent. Personal data of the participants were not registered at any stage of the study. Participation in this study was voluntary and anonymous. The previous engagement of the first author in the Deaf community, as she has a National Certification in GSL and participates in their community activities, reinforced the feelings of trust and comfort of the participants, leading to a high response rate (91%). The response rate for the hearing participants was 93%.

### Statistical analysis

Categorical variables are presented as absolute (n) and relative (%) frequencies, while quantitative variables are presented as mean (standard deviation, SD) or median (range). The Kolmogorov-Smirnov test and normal plots were used to test the normality of quantitative variable distribution.

Firstly, we performed bivariate analyses between socio-demographic and non-medical characteristics and scores on SF-32v2 scales. Bivariate associations between categorical variables were assessed with chi-square test, while between categorical and ordinal variables with chi-square trend test. Student’s t-test and analysis of variance were used for the association between categorical variables and a continuous one that followed normal distribution. Also, we used Kruskal-Wallis test to assess the relation between categorical variables and a continuous one that did not follow normal distribution. We used Pearson’s and Spearman’s correlation coefficient to find out the correlation between continuous variables that followed and did not follow normal distribution respectively.

Scores on SF-32v2 scales were the dependent variables. In case that > 2 independent variables were associated with scores on SF-32v2 scales (*p* < 0.2) in bivariate analyses, then multivariate linear regression analysis was applied, using the backward stepwise linear regression model in order to eliminate confounding. For multivariate linear regression models, coefficients’ beta values, 95% confidence intervals and *p* values are presented.

‘Work status’ was not used as an independent variable due to the small number of participants in several categories (*n* ≤ 10).

The five designed subcategories of population with hearing loss were not finally used as independent variables due to the exceptionally small number of participants in several categories (n ≤ 10). Therefore, the classification was limited to the three bigger categories (deaf/ hard of hearing/hearing) and it was not possible to consider the exact language preferences of the participants, but only their cultural self-identification. That means that the “deaf category” in the statistical analysis finally included those that self-identified themselves as severely or profoundly deaf and used to communicate via GSL or non-signing communication (category No1 and No2), while the “hard of hearing category” included those that self-identified themselves as having mild, moderate or moderately severe hearing loss and used to communicate in terms of auditory-verbal approach or via lip-reading (category No3 and No4).

The two-tailed significance level was set ≤0.05. Data were analyzed using IBM SPSS 21.0 (IBM Corp. Released 2012. IBM SPSS Statistics for Windows, Version 21.0. Armonk, NY: IBM Corp).

## Results

### Socio-demographic characteristics

Socio-demographic characteristics are presented in Table 1. The two-tailed significance level was set ≤0.05. For the 91.9% of deaf (*n* = 79) the GSL was the preferred method of communication, while for the 8.1% (n = 7) was not. Among the 54 hard of hearing, the 20.4% (*n* = 11) preferred to communicate via the oral method, the 50% (*n* = 27) via the lip-reading technique and the 29.6% (*n* = 16) via the GSL.

**Table 1:** Participants socio-demographic characteristics, *N = 237*

Variable	Hearing	Hard of Hearing	Deaf	*P*-value
Gender				0.02*^a^
Women	71 (73.2)	32 (59.3)	46 (53.5)	
Men	26 (26.8)	22 (40.7)	40 (46.5)	
Age (years) ^b^	36.3 (9.4)	41.4 (11.8)	38.1 (10.7)	0.02*^c^
Marital Status				0.006**^a^
Unmarried	42 (43.3)	16 (29.6)	37 (43.0)	
In cohabitation	13 (13.4)	14 (25.9)	11 (12.8)	
Married	38 (39.2)	14 (25.9)	20 (23.3)	
Divorced	3 (3.1)	7 (13.0)	13 (15.1)	
Widowed	1 (1.0)	3 (5.6)	5 (5.8)	
Number of family members per household ^e^	3.5 (1.3)	2.7 (1.2)	2.6 (1.2)	< 0.001***^c^
Existence of hearing person in household ^a^		38 (70.4)	37 (43)	0.002**^a^
Educational attainment				< 0.001***^d^
Junior High	0 (0.0)	4 (7.4)	11 (12.8)	
High School	23 (23.7)	19 (35.2)	59 (68.6)	
College	9 (9.3)	3 (5.6)	2 (2.3)	
Technological Educational Institution (TEI)	13 (13.4)	5 (9.3)	0 (0)	
University	31 (32.0)	11 (20.4)	10 (11.6)	
Master’s/Doctorate degree	21 (21.6)	12 (22.2)	4 (4.7)	
Work Status				0.001**^a^
Unemployed	10 (10.3)	8 (14.8)	21 (24.4)	
Household keeper	2 (2.1)	3 (5.6)	5 (5.8)	
Income collection	2 (2.1)	0 (0)	1 (1.2)	
Student	10 (10.3)	1 (1.9)	5 (5.8)	
Unskilled worker	0 (0)	4 (7.4)	6 (7.0)	
Private sector employee	31 (32.0)	10 (18.5)	30 (34.9)	
Public sector employee	27 (27.8)	18 (33.3)	16 (18.6)	
Entrepreneur	12 (12.4)	6 (11.1)	0 (0)	
Retired	3 (3.1)	4 (7.4)	2 (2.3)	
Family annual income (euro) ^e^	20,000 (195,000)	15,000 (75,000)	15,000 (49,000)	< 0.001***^f^

Values are expressed as n (%) unless otherwise is indicated

*p value 0.01 to 0.05 (significant)

**p value 0.001 to 0.01 (very significant)

***p value 0.0001 to 0.001 (extremely significant)

^a^x^2^ test

^b^Mean (standard deviation)

^c^Analysis of variance

^d^x^2^ trend test

^e^Median (range)

^f^Kruskal-Wallis test

### Non-medical determinants of health

Non-medical determinants of health are shown in Table 2. The mean of BMI was higher for deaf compared to hard of hearing and hearing people (*p* = 0.02). There was a higher proportion of overweight (55.8%) and obese people (20.9%) among deaf and hard of hearing adults (55.6% and 18.5% accordingly), while almost half of the hearing participants (44.3%) had a normal BMI (*p* = 0.004) (Table 2).

**Table 2:** Participants’ non-medical determinants of health, *N = 237*

Variable	Hearing	Hard of Hearing	Deaf	P-value
Tobacco consumer				0.13^a^
Current	38 (39.2)	20 (37.0)	48 (55.8)	
Former	18 (18.6)	9 (16.7)	10 (11.6)	
Never	41 (42.3)	9 (46.3)	28 (32.6)	
Number of cigarettes smoked per day ^e^	11 (59)	20 (58)	20 (35)	< 0.001***^f^
Years of smoking ^e^	12 (37)	20 (43)	18 (39)	0.01*^f^
Alcohol consumption (units of alcohol per week) ^e^	1 (10)	2 (10)	3.5 (15)	< 0.001***^f^
Body Mass Index (BMI) ^b^	26.1 (4.6)	27.4 (4.2)	27.9 (4.1)	0.02*^c^
BMI Classification				0.004**^d^
Underweight	2 (2.1)	0 (0)	0 (0)	
Normal	43 (44.3)	14 (25.9)	20 (23.3)	
Overweight	37 (38.1)	30 (55.6)	48 (55.8)	
Obese	15 (15.5)	10 (18.5)	18 (20.9)	
Physical activity (hours per week) ^e^	2 (10)	2 (10)	0 (8)	< 0.001***^f^

Values are expressed as n (%) unless otherwise is indicated

*p value 0.01 to 0.05 (significant)

**p value 0.001 to 0.01 (very significant)

***p value 0.0001 to 0.001 (extremely significant)

^a^x^2^ test

^b^Mean (standard deviation)

^c^Analysis of variance

^d^x^2^ trend test

^e^Median (range)

^f^Kruskal-Wallis

Concerning tobacco consumption, the median number of cigarettes smoked per day was higher for hard of hearing and deaf compared to the hearing participants (*p* < 0.001). Besides, the median number of years of smoking was higher for hard of hearing and deaf compared to the hearing participants (*p* = 0.01).

The median hours of physical activity per week was higher for hearing and hard of hearing compared to deaf participants (p < 0.001). The median number of units of alcohol per week was higher for deaf compared to hard of hearing and hearing participants (p < 0.001).

### Short-form health survey

Cronbach’s coefficient alpha across the domains of SF-36v2 ranging from 0.91 to 0.93, indicating high internal consistency reliability.

The bivariate associations between all independent variables and scores on SF-32v2 scales are presented in Table 3. In case that > 2 independent variables were associated with scores on SF-32v2 scales (*p* < 0.2) in bivariate analyses, then multivariate linear regression analysis was applied, using the backward stepwise linear regression model in order to eliminate confounding.

**Table 3:** Bivariate associations between independent variables and mean scores on SF-32v2 scales (Standard Deviation), *N = 237*

Variable	Physical Functioning	Role-Physical	Bodily Pain	General Health	Vitality	Social Functioning	Role-Emotional	Mental Health	Physical component summary score	Mental component summary score
Gender ^a^										
Women	49,3 (6,9)	46,9 (9,1)	48,9 (10,5)	49,0 (9,7)	46,2 (10,5)	44,6 (9,5)*	41,9 (10,2)	40,5 (11,0)	51,6 (6,8)	40,1 (10,8)
Men	49,4 (9,6)	46,1 (10,1)	47,2 (10,5)	47,8 (12,1)	46,6 (12,9)	41,6 (10,9)*	40,6 (11,7)	39,0 (13,1)	51,1 (8,9)	38,5 (12,7)
Age (years) ^b^	−0,24*	− 0,13*	− 0,13*	−0,16*	− 0,01	− 0,06	− 0,05	− 0,05	− 0,24*	− 0,001
BMI ^b^	−0,39*	− 0,28*	− 0,14*	−0,23*	− 0,22*	− 0,29*	− 0,21*	−0,25*	− 0,30*	−0,22*
Hearing status ^c^										
Normal hearing	52,9 (5,8)*	52,6 (5,9)*	51,7 (9,2)*	52,4 (8,1)*	50,6 (10,4)*	47,7 (9,2)*	46,5 (8,5)*	43,3 (9,4)*	55,5 (5,0)*	43,6 (9,8)*
Hard of hearing	49,4 (7,5)*	46,7 (8,2)*	48,2 (9,9)*	48,5 (10,9)*	49,0 (11,6)*	44,7 (8,9)*	43,2 (9,1)*	41,9 (11,8)*	50,8 (7,6)*	42,2 (10,7)*
Deaf	45,3 (8,5)*	39,8 (8,9)*	44,5 (11,1)*	44,3 (11,5)*	39,7 (9,1)*	37,9 (9,4)*	34,5 (10,4)*	34,9 (12,7)*	47,4 (7,8)*	33,2 (11,3)*
Marital Status ^c^										
Category 1 (unmarried, divorced, widowed)	48,6 (8,3)*	45,6 (9,2)*	48,6 (10,6)	47,5 (10,4)*	45,2 (10,7)*	42,7 (9,8)*	40,4 (10,7)*	38,7 (11,5)*	51,1 (7,6)	38,2 (11,1)*
Category 2 (married, in cohabitation)	50,1 (7,6)*	47,8 (9,6)*	47,9 (10,5)	49,8 (10,7)*	47,6 (12,1)*	44,5 (10,4)*	42,6 (10,7)*	41,4 (12,1)*	51,9 (7,8)	41,0 (11,9)*
Number of family members per household ^b^	0,18*	0,17*	0,07	0,20*	0,21*	0,16*	0,17*	0,21*	0,14*	0,20*
Educational attainment ^d^	0,43*	0,43*	0,27*	0,32*	0,29*	0,32*	0,34*	0,23*	0,43*	0,25*
Family annual income (euro) ^d^	0,15*	0,28*	0,23*	0,21*	0,27*	0,37*	0,36*	0,32*	0,16*	0,37*
Tobacco consumer ^c^										
Current	47,8 (8,3)*	44,8 (9,3)*	48,6 (11,0)	46,7 (10,7)*	44,5 (11,8)*	42,5 (9,5)	40,3 (10,7)	39,4 (12,5)	50,1 (8,0)*	50,1 (8,0)*
Former	50,0 (8,2)*	48,2 (9,8)*	47,4 (10,8)	50,0 (11,2)*	47,2 (12,1)*	44,6 (10,4)	42,1 (11,7)	41,1 (13,1)	52,0 (7,3)*	52,0 (7,3)*
Never	50,8 (7,4)*	48,0 (9,2)*	48,3 (9,9)	50,0 (10,2)*	48,0 (10,4)*	44,2 (10,7)	42,4 (10,4)	40,0 (10,5)	52,8 (7,1)*	52,8 (7,1)*
Physical activity (hours per week) ^d, e^	0,52*	0,29*	0,06	0,35*	0,28*	0,22*	0,25*	0,18*	0,37*	0,19*
Alcohol consumption (units of alcohol per week) ^d, e^	−0,31*	− 0,37*	−0,22^*^	− 0,23*	−0,31*	− 0,23*	− 0,33*	− 0,27*	− 0,29*	− 0,29*

Values are expressed as n (%) unless otherwise is indicated

*p < 0.20

^a^t- test, ^b^ Pearson correlation coefficient, ^c^ Analysis of variance, ^d^ Spearman’s rank correlation coefficient, ^e^ Median (range)

Multivariate linear regression analyses revealed a statistically significant relation between hearing loss and lower SF-36v2 scores across subscales, even after controlling for confounders. If the confidence interval did not include the value of zero effect, it was assumed that there was a statistically significant result (Table 4). Concerning the physical health domain, deaf participants had a lower Physical Functioning score compared to hearing, a lower Role Physical Score compared to hearing and hard of hearing, a lower Bodily Pain score compared to hearing, and a lower Physical Health Component Summary score compared to hearing. Concerning the physical health domain, deaf participants had a lower Vitality score compared to hearing and hard of hearing, a lower Social Functioning score compared to hearing and hard of hearing, a lower Role Emotional score compared to hearing and hard of hearing, a lower Mental Health score compared to hearing and hard of hearing, and a lower Mental Health Component Summary score compared to hearing and hard of hearing.

**Table 4:** Multivariate linear regression analysis with SF-36v2 domains’ scores as dependent variables

	b coefficient (95% CI)	*P*-value
Physical Functioning		
BMI	−0.58 (− 0.76 to − 0.40)	< 0.001
Hearing compared to deaf	2.06 (0.29 to 3.83)	0.023
Educational level	1.05 (0.53 to 1.58)	< 0.001
Physical Activity (hours per week)	1.09 (0.73 to 1.44)	< 0.001
Alcohol consumption (units of alcohol per week)	−0.37 (−0.69 to − 0.05)	0.023
Role Physical		
BMI	−0.40 (− 0.62 to − 0.19)	< 0.001
Hearing compared to deaf	9.52 (6.95 to 12.09)	< 0.001
Hard of hearing compared to deaf	4.51 (1.86 to 7.17)	0.001
Educational level	0.88 (0.23 to 1.52)	0.008
Alcohol consumption (units of alcohol per week)	−0.45 (−0.84 to − 0.05)	0.027
Bodily Pain		
Hearing compared to deaf	3.18 (0.31 to 6.05)	0.03
Alcohol consumption (units of alcohol per week)	−0.78 (− 1.30 to − 0.26)	0.004
General Health		
BMI	−0.44 (− 0.71 to − 0.16)	0.002
Educational level	0.91 (0.11 to 1.70)	0.024
Physical Activity (hours per week)	−0.96 (−1.44 to − 0.48)	< 0.001
Alcohol consumption (units of alcohol per week)	1.05 (0.49 to 1.61)	< 0.001
Vitality		
BMI	−0.40 (− 0.70 to − 0.10)	0.009
Hearing compared to deaf	7.86 (4.48 to 11.22)	< 0.001
Hard of hearing compared to deaf	7.45 (3.86 to 11.09)	< 0.001
Alcohol consumption (units of alcohol per week)	−0.55 (−1.08 to − 0.03)	0.039
Social Functioning		
BMI	−0.52 (− 0.78 to − 0.26)	< 0.001
Hearing compared to deaf	7.45 (4.50 to 10.40)	< 0.001
Hard of hearing compared to deaf	5.54 (2.37 to 8.72)	0.001
Educational level	0.78 (0.03 to 1.54)	0.043
Role Emotional		
BMI	−0.32 (−0.58 to − 0.05)	0.022
Hearing compared to deaf	9.04 (5.99 to 12.10)	< 0.001
Hard of hearing compared to deaf	6.80 (3.52 to 10.07)	< 0.001
Alcohol consumption (units of alcohol per week)	−0.60 (−1.08 to − 0.13)	0.013
Physical Activity (hours per week)	0.55 (0.01 to 1.09)	0.046
Mental Health		
BMI	−0.54 (− 0.86 to − 0.22)	0.001
Hearing compared to deaf	4.79 (1.24 to 8.34)	0.008
Hard of hearing compared to deaf	5.02 (1.20 to 8.84)	0.010
Alcohol consumption (units of alcohol per week)	−0.97 (−1.53 to − 0.41)	0.001
	b coefficient (95% CI)	Test (*p*-value)
Physical Health Component Summary Score		
BMI	−0.39 (− 0.57 to − 0.21)	< 0.001
Hearing compared to deaf	3.87 (2.05 to 5.68)	< 0.001
Educational level	1.07 (0.54 to1.59)	< 0.001
Alcohol consumption (units of alcohol per week)	−0.51 (− 0.83 to 0.18)	0.002
Physical Activity (hours per week)	0.64 (0.28 to 0.99)	0.001
Number of family members per household	−0.74 (−1.39 to − 0.09)	0.026
Mental Health Component Summary Score		
BMI	−0.43 (− 0.73 to 0.12)	0.006
Hearing compared to deaf	7.62 (4.21 to 11.02)	< 0.001
Hard of hearing compared to deaf	7.52 (3.86 to 11.17)	< 0.001
Alcohol consumption (units of alcohol per week)	−0.70 (−1.24 to − 0.17)	0.010

Respondents with a higher degree of hearing loss had lower scores in all SF-36 sections, excluding the General Health score, while decreased body mass index (BMI) was associated with increased scores in all SF-36 sections, excluding the Bodily Pain score.

Higher educational level was associated with increased scores in Physical Functioning, Role Physical, Bodily Pain, General Health, Social Functioning and Physical Health Component Summary scores and increased physical activity was associated with increased scores in Physical Functioning, General Health, Role Emotional and Physical Health Component Summary scores.

Finally, decreased alcohol consumption was associated with increased scores in all the SF-36v2 dimensions except social functioning and decreased number of family members per household was associated with increased Physical Health Component Summary score.

## Discussion

Previous studies have revealed that disabling hearing loss, which refers to hearing loss greater than 41 dB in the better hearing ear in adults, is associated with poorer physical health [19–23] and lower levels of mental health [24–34]. This study adds to the limited number of studies which associated the degree of hearing loss with poorer HRQoL in young and middle-aged adults [5, 7, 9–11, 32], while according to our knowledge it is the only one where the HRQoL was evaluated via the 36-Item Short Form Health Survey (SF-36) in this specific population, and did not concern older adults [35, 36]. The findings of our study showed that respondents with higher degrees of hearing loss had lower scores in physical functioning, role physical, bodily pain, vitality, social functioning, role emotional, mental health domains, Physical Health and Mental Health Component.

Decreased numbers of household individuals, and particularly individuals living alone, has been associated with lower mental health levels, which is associated with depression among adults with hearing loss [31, 34]. However, in our study, decreased numbers of household individuals was associated with increased Physical Health Component Summary score. One possible explanation for this paradox is the better ability of participants to pay privately for health and interpreting services if a smaller number of persons cohabiting shares a low household’s income. This may happen as the very high percentage of private payments is a significant characteristic of the mixed financial resources of the Greek health care system [37].

The findings of our study showed that increased education was associated with increased scores in Physical Functioning, Role Physical, Bodily Pain, General Health, Social Functioning and Physical Health Component Summary and are in compliance with previous studies [10, 19, 21, 36, 38]. McKee et al. [38], have also supported that educational attainment is more important than higher income as a physical health protecting factor.

Concerning the variable tobacco consumption, in this study the 55.8% of deaf were current tobacco consumers, while in the Health Interview Survey [39] the rate for the general population was 32.5%. The median number of cigarettes smoked per day was higher in hard of hearing and deaf compared to hearing adults, but the multivariate linear regression did not reveal a statistically significant correlation between the tobacco consumption and health-related quality of life. Conversely, Schoenborn & Heyman [20], had revealed that adults with lower or higher level of hearing loss used to smoke at higher rates than the hearing population (40% instead of 24%), while other studies [19, 22, 23] have shown lower rates of tobacco consumption among deaf or hard of hearing compared to hearing individuals. In a previous study [34], tobacco consumption (current/former tobacco consumer) has been associated with increased risk to mental health problems, while is associated with depression among adults with hearing loss.

Also, we found that decreased alcohol consumption was associated with increased scores in all the SF-36v2 dimensions except social functioning. The higher number of alcohol units per week has been associated also in a previous study [34] with lower mental health levels, which is associated with depression among adults with hearing loss [31, 34].

Respectively, we found that 55.8% and 20.9% of deaf were overweight and obese, while the percentages in hearing people were 38.1% and 15.5%. In the Health Interview Survey [39], percentages in the general population were 39.4% and 16.9%. In addition, we found that decreased body mass index was associated with increased scores on all SF-32v2 scales, except for the bodily pain score. Weight that is *higher than* what is considered as a *healthy* weight, has been associated also in previous studies with lower level of health-related quality of life, in both physical and mental health domains for population with hearing loss [19, 20, 22, 23, 34].

Also, in our study, increased physical activity was associated with increased scores in physical functioning, general health, role emotional and physical health component summary. In previous studies the lower physical activity levels have also been associated with population with hearing loss [19, 20].

### Limitations

There are several limitations when conducting a study among deaf and hard of hearing participants. However, the previous engagement of the first author (TD) with the Deaf community, allowed many of these methodological issues being eliminated as much as possible.

The main limitation of our study is that we did not use an additional specific questionnaire for hearing loss – for example the Hearing Handicap Inventory for Adults (HHIA) – to identify problems that hearing loss may cause to participants, as there have been expressed doubts in literature concerning differential performance of SF-36 items in healthy adults with and without functional limitations [40]. However, this multidimensional 36-item Health Survey has been used widely across a range of diseases and treatment groups. Even if certain questions may not intuitively relate to hearing impairment, it has been argued that allows the direct comparison of the effects of several determinants including hearing impairment on the health-related quality of life of populations [35]. Moreover, we considered the SF-36v2 sufficient as it has been translated and its psychometric properties have been tested for a Greek population [18], while the Hearing Handicap Inventory for Adults (HHIA) has not. Another limitation was that the deaf population consists of subgroups with different cultural and communicational characteristics, which need to be separately examined. In this study, the five designed subcategories of population with hearing loss were not used as independent variables. The reason was the exceptionally small number of participants in several categories (*n* ≤ 10). As a result, the classification was limited to the three bigger categories (deaf/ hard of hearing/hearing) and it was not possible to take into consideration the language preferences of the participants, but only their cultural self-identification. This should be considered by future studies aiming to assess the health-related quality of life in a larger sample of population with hearing loss, as e.g. the Deaf community struggles with a number of socioeconomic-based health disparities, many of which are not directly associated with the degree of hearing loss.

Validating and standardizing a conceptual model [11] of health-related quality of life among people with a range of functional hearing and language preferences, including GSL, that delineates the relationships between health status (self-acceptance, coping with limitations), intrinsic (functional communication skills, navigating barriers/self-advocacy, resilience) and extrinsic (acceptance by others, access to information, educating others) factors in their influence on quality of life outcomes among young and middle-aged adults with hearing loss, should be considered for the future.

## Conclusions

As hearing loss is an important global health concern, health-related quality of life among deaf and hard of hearing adults must be estimated as a factor of great importance. In Greece, people with hearing disabilities are systematically excluded in health policy and planning processes, despite there is a marked tendency for global efforts aimed at improving their quality of life.

Identifying determinants of the health- related quality of life of deaf and hard of hearing people is decisive, so as their health condition can be improved. The findings of this study suggest that hearing loss is capable of contributing to HRQoL deficits in Greek deaf and hard of hearing adults, as their health is poorer than that of the general population, with probable underdiagnosis and undertreatment of chronic conditions, putting them at risk of preventable ill health.

Health information is not available in sign language for Deaf people and it may affect their skills in taking decisions in everyday life concerning health promotion. That is being reflected in several determinants (such as tobacco and alcohol consumption, BMI and physical activity). Programs that raise health knowledge in Deaf communities could contribute to improve their skills in take decisions in everyday life concerning the promotion of a healthy lifestyle. This could help in tackling health inequalities for people with hearing loss and in improving their quality of life.

Future research should involve a more extensive population with hearing loss, in order to produce health statistics of deaf and hard of hearing populations, so as they can be included in the priorities for improving health and chronic disease prevention programs and stop therefore this health condition from being a “silent epidemic” [4].

## Abbreviations

GSL: Greek sign language
